# Adaptation and validation of the Intrinsic Motivation Inventory (IMI) in Polish

**DOI:** 10.3389/fpsyg.2026.1822953

**Published:** 2026-06-10

**Authors:** Jolanta Enko, Maciej Kościelniak

**Affiliations:** Faculty of Psychology and Law, SWPS University, Poznań, Poland

**Keywords:** bilingual assessment, cross-language invariance, CT-C(M-1), intrinsic motivation, intrinsic motivation inventory (IMI), psychometric validation, scale adaptation, self-determination theory

## Abstract

**Introduction:**

The aim of this study was to adapt the full 45-item, seven-subscale Intrinsic Motivation Inventory (IMI) into Polish and evaluate its psychometric properties, including cross-language measurement invariance in Polish–English bilinguals.

**Methods:**

Two independent online samples were pooled (*N* = 275), with participants completing the IMI in both Polish and English. Reliability (*α*, *ω*), cross-language correlations, and confirmatory factor analyses (CFA; WLSMV/THETA) were conducted, while language invariance was tested using a CT-C(M–1) framework comparing marker-configural and strict metric models. Convergent and divergent validity were assessed through associations with external constructs related to motivation, control, curiosity, and stress appraisal.

**Results:**

CFA supported the expected seven-factor structure in both languages with good model fit, and language invariance analysis indicated minimal configural–metric differences, suggesting approximate measurement invariance. The Polish IMI demonstrated strong internal consistency across all subscales, closely matching the English version, while cross-language correlations confirmed strong subscale-level equivalence, though a small number of items showed weaker psychometric properties. Subscale intercorrelations followed theoretical expectations, supporting construct validity, with subscales reflecting engagement, autonomy, and competence aligning most strongly with positive motivational constructs. Overall, the Polish IMI is suitable for research in Polish, with all materials, data, and analysis code openly available.

## 1 Introduction

One of the most widely used instruments for assessing intrinsic motivation is the Intrinsic Motivation Inventory (IMI; Ryan, 1982; Ryan et al., 1983; McAuley et al., 1989), grounded in Self-Determination Theory (SDT). The IMI provides a multidimensional assessment of subjective motivational experience, capturing both core intrinsic motivation and related constructs such as perceived competence, autonomy, and task value. Despite widespread international use, no formally validated Polish version of the IMI currently exists. While other Polish-language instruments measuring intrinsic motivation are available—such as the Academic Motivation Scale (Ardeńska et al., 2019), the Work Extrinsic and Intrinsic Motivation Scale (Chrupała-Pniak and Grabowski, 2016), and the Elementary School Motivation Scale (Nikel, 2021)—they do not fully capture the same motivational dimensions assessed by the IMI and are tailored to specific fields. The absence of a validated Polish IMI thus limits researchers’ ability to conduct methodologically sound studies aligned with SDT in this linguistic and cultural context.

Within SDT, intrinsic motivation is understood as engagement driven by interest, enjoyment, and inherent satisfaction, and is closely tied to the fulfillment of basic autonomy, competence and relatedness needs (Ryan and Deci, 2000; Deci and Ryan, 2008; Ryan and Deci, 2020). The full IMI consists of seven subscales assessing not only intrinsic motivation itself but also related constructs that contribute to the overall motivational experience.

The inventory was originally developed to evaluate participants’ subjective experiences related to specific activities or tasks in experimental contexts (Ryan, 1982; McAuley et al., 1989). The subscales can be used collectively or selectively, depending on the study goal. The item wordings are adaptable to specific contexts by substituting the name of the activity or task. The IMI produces a profile across subscales, providing nuanced information about motivational dynamics. The seven subscales are:

- Interest/Enjoyment – primary indicator of intrinsic motivation;
- Perceived Competence – sense of efficacy during the activity;
- Effort/Importance – how much effort is invested;
- Pressure/Tension – pressure or anxiety;
- Perceived Choice – whether behavior is autonomous;
- Value/Usefulness – perceived utility of the activity;
- Relatedness – social connectedness in the activity.

The original validation study by McAuley et al. (1989) supported a five-factor model with second-order factor representing general intrinsic motivation. However, later adaptations have proposed alternative models or emphasized partial invariance across contexts (e.g., Monteiro et al., 2015). Various adaptations (e.g., German, Wilde et al., 2009; Turkish, Duman et al., 2020; Austrian, Cocca et al., 2022, Brazilian Portuguese, de Azevedo et al., 2019; Nunes and Darin, 2023) also reveal variations in factor structure and item performance across cultural and contextual settings. Many utilized only part of the original questionnaire, and most are tailored to specific fields such as education, sports, or health psychology. Such reductions often improve model fit artificially, but the conceptual coverage of IMI is altered. Full-form cross-language equivalence tested on within-person bilingual design (as opposed to using independent language groups) allows for better tests of measurement equivalence, separating trait variance from language-specific variance. The present study therefore focuses on the full 45-item version to preserve comparability with the original multidimensional structure and to test its robustness.

We provide preliminary construct validation through correlations between IMI subscales and related measures. Because participants were instructed to reflect on a self-selected collaborative activity that could occur in different domains (e.g., work or university), existing Polish domain-specific intrinsic motivation scales (e.g., work-only or school-only measures) would not have been aligned with the task context. Moreover, the IMI captures situational, task-level motivational experiences. In contrast, other available intrinsic motivation scales assess more stable, trait-like orientations within specific domains. Using a trait-level measure as a criterion for a situational construct would introduce a mismatch in construct level, potentially obscuring rather than clarifying validity evidence. Construct validity was therefore evaluated through theoretically adjacent constructs operating at a comparable situational level (e.g., attitudes toward the task, challenge appraisal, perceived control), which provide conceptually appropriate anchors for task-specific intrinsic motivation. At present, no validated domain-general task-specific intrinsic motivation measure is available in Polish that would allow for a direct parallel comparison. This need for a domain-general, task-level measure of intrinsic motivation is precisely the rationale for adapting the IMI to the Polish context.

Based on Self-Determination Theory and related motivational frameworks, the IMI subscales were expected to demonstrate theoretically coherent patterns of association with conceptually related constructs (see Table 1). Subscales reflecting intrinsic engagement (Interest/Enjoyment, Effort/Importance, Value/Usefulness) were expected to correlate positively with favorable task attitudes, challenge appraisal, and curiosity-related tendencies. Competence- and autonomy-related subscales were expected to correlate positively with perceived behavioral control and general self-efficacy, reflecting overlaps in agency and self-determination, and negatively with threat appraisal. In contrast, Pressure/Tension was expected to show the opposite pattern, displaying negative associations with adaptive motivational constructs and positive associations with threat.

**Table 1:** Expected construct validity patterns for Polish IMI subscales.

IMI subscale	Expected strong positive correlations	Expected moderate correlations	Expected weak/negative correlations
Interest/enjoyment	Attitude toward task	Challenge appraisal, curiosity	Threat appraisal (negative)
Perceived competence	Perceived behavioral control, Self-efficacy	Attitude, challenge appraisal	Threat appraisal (negative)
Effort/importance	Attitude toward task	Challenge appraisal	—
Pressure/tension	Threat appraisal	—	Attitude, perceived control, curiosity, self-efficacy (all negative)
Perceived choice	Perceived behavioral control	Attitude, challenge, curiosity, self-efficacy	Threat appraisal (negative)
Value/usefulness	Attitude toward task	Perceived control, challenge, curiosity, self-efficacy	Threat appraisal (negative)
Relatedness	Attitude toward task	—	Threat appraisal (negative)

The present study therefore provides a full Polish adaptation of the IMI, preserving its original seven-factor structure and ensuring comparability with prior Self-Determination Theory research. Beyond translation, we evaluate its factorial validity, reliability, and cross-language equivalence using a within-person bilingual design. In contrast to traditional independent-group approaches to measurement invariance, this design enables a more stringent test of equivalence by modeling language as a method factor within a multitrait–multimethod (CT-C[M–1]) framework. This approach allows trait variance to be disentangled from language-related method effects, thereby clarifying the extent to which observed differences reflect substantive constructs rather than linguistic artifacts. By combining full-form validation with within-person cross-language modeling, the study addresses both the substantive need for a domain-general, task-level measure of intrinsic motivation in Polish and the methodological challenge of establishing equivalence across language versions.

## 2 Materials and methods

### 2.1 Participants

The target sample size was 200 participants based on feasibility constraints; no formal *a priori* power analysis was conducted. A total of 198 participants (*M_*age = 26.7 years, *SD* = 8.09 years; 47% women) were recruited through the Prolific platform (prolific.co). Participants received compensation of approximately £2.10 for completing the study. Inclusion criteria included Polish–English bilingualism. Participants self-reporting A1–A2 English proficiency were excluded from analyses involving English items. After exclusion, 181 participants remained (*M_*age = 26.50 years, *SD* = 7.60 years; 47.5% women). All participants passed the Attention Check (a question asking them to choose a specific answer). The study was conducted online using the Qualtrics survey platform. This group is labeled as S1 in Results.

To assess the stability of the findings, an additional independent sample of 94 participants (excluding A1–A2 English proficiency) was collected using the same platform and procedure (*M_*age = 28.30 years, *SD* = 8.14 years; 46.8% women) and served as internal replication (labeled S2 in Results). As both samples completed the same measures under equivalent conditions, and measurement invariance was supported across samples, responses from both samples were pooled for the primary analyses (*N* = 275; *M_*age = 27.10 years, *SD* = 7.82 years; 47% women).

Participants were predominantly residents of Poland, with only a small number residing in other countries. As such, the sample reflects a population typical of online research panels, consisting largely of young, digitally engaged adults recruited via Prolific.

### 2.2 Procedure

#### 2.2.1 Preparation of the Polish version

Permission to adapt the Intrinsic Motivation Inventory (IMI) into Polish was obtained from the original authors. Three independent forward translations were prepared by bilingual psychologists. These were reviewed and integrated into a preliminary Polish version. An independent back-translation was then prepared by a separate translator and compared with the original to identify any meaning discrepancies. The final version was reviewed by a professional Polish linguist.

#### 2.2.2 Validation of the Polish version

Participants were invited to reflect on a recent social task or activity completed collaboratively with someone they did not know well, either at work or university. They were instructed to briefly describe this task and to refer to it consistently throughout all questionnaire responses (IMI and scales for examining construct validity). The order of questionnaire versions (Polish vs. English) and the order of items within each version were randomized to control for order effects. The instructions were as follows: “Think of a task or activity you recently completed at work or university together with someone you did not know well. Briefly describe this activity in one or two sentences. Throughout the entire study, when answering questions about a task or activity, refer to the one you described here.”

### 2.3 Measures

Intrinsic Motivation Inventory (IMI) (Ryan, 1982; Ryan et al., 1983): The instrument comprises 45 items across seven subscales, rated on a 7-point scale (from “not at all true” to “very true”). Reliability estimates for each language version are reported in the Results section.

#### 2.3.1 Scales for construct validity

Theory of Planned Behavior components: Task-specific attitudes and perceived behavioral control, based on Ajzen’s framework (Francis et al., 2004); Polish version by Kaczmarek et al. (2015). Attitudes are measured on three 7-point bipolar adjective scales (*α* = 0.81, *ω* = 0.83), and perceived control on three items on a 7-point scale from “completely disagree” to “completely agree” (*α* = 0.87, *ω* = 0.88).

#### 2.3.2 Curiosity and exploration inventory–II (CEI-II)

Assesses trait curiosity (Kashdan et al., 2009; Polish adaptation by Kaczmarek et al., 2013) on 10 items with 5-point scale from 1 “very slightly or not at all” to 5 “extremely” (*α* = 0.91, *ω* = 0.91).

#### 2.3.3 Cognitive appraisal scale (CAS)

Measures cognitive evaluations of challenging situations (Skinner and Brewer, 2002); Polish adaptation by Enko et al. (2021). It consists of two subscales measuring challenge and threat. Participants answered on a 6-point scale ranging from “I strongly disagree” to “I strongly agree” (α = 0.79, ω = 0.80 for challenge and α = 0.94, ω = 0.94 for threat).

#### 2.3.4 General self-efficacy scale (GSES)

Assesses beliefs in one’s ability to cope with a variety of situations (Schwarzer and Jerusalem, 1995; Polish version by Juczyński, 2000). Participants answered 10 items on a 4-point scale (1 = Not at all true, 2 = Hardly true, 3 = Moderately true, 4 = Exactly true) (α = 0.89, ω = 0.89).

### 2.4 Ethical approval and informed consent

The study was reviewed and approved by the department’s Institutional Ethics Committee (Approval no. 2023-189, issued on May 23, 2023). All participants provided informed consent before participation.

### 2.5 Statistical analyses

All primary analyses were conducted on the pooled dataset (*N* = 275). Separate sample-specific data, codes and outputs are available on OSF.

#### 2.5.1 Confirmatory factor analysis

Confirmatory factor analyses (CFA) with WLSMV estimator and THETA parametrization were first used to test the hypothesized seven-factor structure of the Intrinsic Motivation Inventory (IMI) separately in Polish and in English. Each subscale was modeled as a latent factor with its intended items as indicators. Although the model is relatively complex (45 items loading on seven factors), simulation studies indicate that sample sizes of N ≥ 200 provide adequate recovery of factor loadings and standard errors under WLSMV (Flora and Curran, 2004; Li, 2016; Liang and Yang, 2014). In the present study, each factor was represented by 5–8 items, which supports estimation stability.

#### 2.5.2 Language invariance

Language invariance was evaluated using a correlated-traits, correlated-(methods-1) CT-C(M–1) model (Eid et al., 2003) estimated with WLSMV/THETA. The Polish version served as the reference method, and an English method factor was specified to capture language-specific variance. Because the Polish and English versions were administered within-person, the two methods are statistically dependent. Traditional multigroup CFA assumes independence between groups and does not explicitly model language-specific method variance. Therefore, a CT-C(M–1) specification was used to disentangle trait variance from language-related method effects. A full CTCM model did not converge, consistent with known identification and parameter-density challenges in large MTMM models. Accordingly, the CT-C(M–1) model was retained. Model fit was compared between configural and metric specifications, and invariance was evaluated based on changes in fit indices (ΔCFI, ΔRMSEA, ΔSRMR). Because the primary aim was to evaluate structural equivalence rather than latent mean differences across languages, scalar constraints were not imposed, as they were not required for the intended interpretations. In addition to the primary CT-C(M–1) model, we explored a conventional repeated-measures CFA framework treating language as two occasions with correlated residuals across language-specific indicators to facilitate comparison with standard invariance approaches. Following estimation of the CFA models using WLSMV, Monte Carlo simulation was conducted to evaluate empirical parameter recovery and convergence based on the fitted population parameters. Simulations were run with 500 replications at sample sizes of 275 and 400 observations per dataset.

#### 2.5.3 Internal consistency and cross-language equivalence

Internal consistency was evaluated via Cronbach’s alpha and McDonald’s omega for each subscale separately. Cross-language equivalence was also assessed through subscale and item level Spearman’s *ρ* correlations and Bayesian estimates of Kendall’s *τ*.

#### 2.5.4 Validity

Convergent and divergent validity were examined via correlations between IMI subscales and external constructs. All correlations were computed using Spearman’s ρ coefficient and Bayesian estimates of Kendall’s τ.

#### 2.5.5 Data analysis and availability

Confirmatory factor analyses were conducted in Mplus 8.11 (Muthén and Muthén, 2017). All other analyses were conducted in jamovi 2.6 (The jamovi project, 2024) or JASP 0.18.3 (JASP Team, 2024).

The datasets analyzed and Mplus, JASP and jamovi files with code for all results for this study can be found on the OSF https://doi.org/10.17605/OSF.IO/CSH38. The full Polish version of IMI is available on Center for Self-Determination Theory website, consistent with how the original English version is distributed.

## 3 Results

### 3.1 Confirmatory factor analysis

The seven-factor model for the Polish IMI demonstrated acceptable fit: *χ*^2^(924) = 3059.33, *p* < 0.001, CFI = 0.919, TLI = 0.913, RMSEA = 0.089 [90% CI: 0.085, 0.092], SRMR = 0.070. The English IMI model also showed comparable fit: *χ*^2^(924) = 2963.21, *p* < 0.001, CFI = 0.910, TLI = 0.904, RMSEA = 0.090 [90% CI: 0.086, 0.093], SRMR = 0.072.

Across both language versions, CFI, TLI, and SRMR indicated acceptable model fit, and the pattern and magnitude of factor loadings and factor correlations were highly similar. Although RMSEA approached conventional cutoffs, fit indices were consistent across languages and supported the intended seven-factor structure. In the Polish CFA, all items loaded significantly on their respective factors (*p* < 0.001). Absolute standardized loadings ranged from 0.528 to 0.956. The pattern of loadings in the English IMI model was similar to the Polish version, with standardized loadings from 0.569 to 0.981 supporting consistency of seven-factor structure across languages. Latent interfactor correlations were moderate in magnitude (|*r*| range = 0.18–0.77) and showed highly similar patterns across Polish and English versions. Taken together, the results indicate cross-language structural consistency of the IMI.

Monte Carlo simulations were conducted for both the Polish and English CFA models (ordinal indicators; WLSMV, theta parameterization) using 275 observations per simulated dataset and 500 replications. Parameter recovery and convergence were evaluated across replications. At *N* = 275, convergence was 58% (290 replications) for the Polish model and 45% (224 replications) for the English model. When the sample size was increased to *N* = 400, convergence improved to 85% (425 replications) (Polish) and 71% (355 replications) (English), consistent with convergence problems at *N* = 275 reflecting finite-sample estimation difficulties with sparse ordinal response patterns under WLSMV rather than structural model misspecification. Across successful replications, the median absolute parameter bias (estimated on converged replications) was approximately 0.04 (Polish) and 0.015 (English) at *N* = 275, and 0.02 (Polish) and 0.01 (English) at *N* = 400. These results suggest that parameter recovery among converged solutions was generally acceptable, but that estimation at the current sample size was not fully stable.

Additional bifactor models were estimated to evaluate whether a general intrinsic motivation factor could account for item covariance beyond the specific IMI subscales. For both Polish and English versions, we tested two specifications: one including a general factor and seven specific subscale factors, and another using Interest/Enjoyment as the reference subscale while estimating the general factor and six remaining specific factors. Across both specifications, bifactor models showed poor fit (Polish: CFI = 0.828–0.835, TLI = 0.814–0.820, RMSEA = 0.128–0.130, SRMR = 0.114; English: CFI = 0.862–0.867, TLI = 0.850–0.855, RMSEA = 0.110–0.112, SRMR = 0.100–0.101). Standardized loadings on the general factor were heterogeneous (absolute ranges: Polish = 0.30–0.93; English = 0.23–0.94). At the same time, specific-factor loadings remained substantial. Thus, neither bifactor specification supported interpreting the IMI as primarily unidimensional, favoring retention of the multidimensional subscale structure.

**Figure 1:**
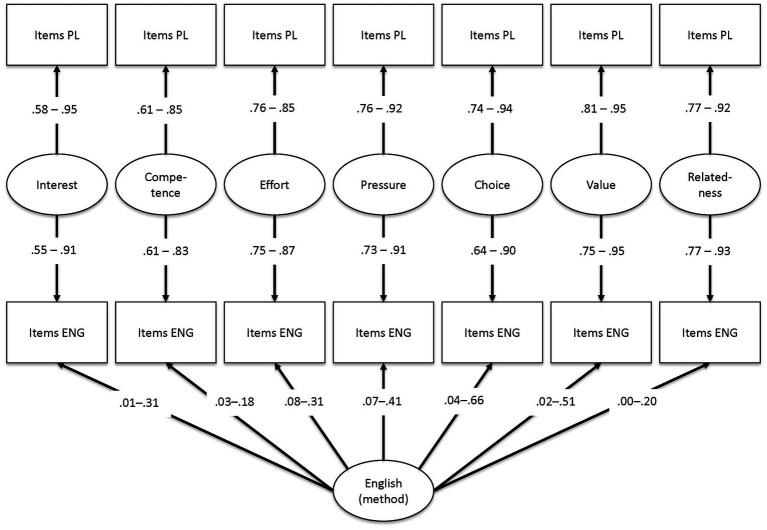
CT-C(M − 1) measurement model with standardized loadings (metric invariance). Ranges represent absolute standardized loadings; signs omitted for clarity. For readability, correlations among trait factors are not displayed. Trait factors were allowed to correlate in the estimated model.

Cross-language measurement invariance was tested using a correlated-traits, correlated-(methods-1) (CT-C(M–1)) model with WLSMV/THETA (Figure 1). Polish indicators served as trait markers, and an English method factor was specified orthogonal to all traits. In the pooled sample, the marker-configural model fit acceptably (CFI = 0.923, RMSEA = 0.062, SRMR = 0.073). Constraining Polish–English loadings to equality on all non-marker pairs (i.e., equating each English item’s trait loading to the corresponding Polish item’s loading) yielded minimal change in approximate fit (CFI = 0.928, RMSEA = 0.060, SRMR = 0.075; ΔCFI = +0.005, ΔRMSEA = −0.002, ΔSRMR = +0.002). However, the DIFFTEST was significant (Δ*χ*^2^(38) = 119.29, *p* < 0.001), indicating that full metric invariance is not strictly supported and that some item loadings may differ across language versions. At the same time, changes in approximate fit indices were within commonly recommended thresholds (|ΔCFI| ≤ 0.010; |ΔRMSEA| ≤ 0.015; Chen, 2007). In the present study, approximate metric invariance is therefore understood as a situation in which equality constraints lead to statistically significant deterioration in exact-fit testing, but only limited changes in fit indices. Given that these guidelines were derived under multigroup specifications, they are used here as approximate descriptive benchmarks rather than definitive criteria. Across both language versions, loadings on the substantive factors were consistently strong (0.551–0.949), whereas loadings on the English method factor were smaller and more variable (approximately 0.000–0.660), indicating that method effects are present but not dominant. Taken together, the results support a cautious interpretation of approximate metric invariance, suggesting that the overall factor structure is broadly comparable across language versions, while allowing for potential item-level differences in loadings.

Exploratory partial invariance analyses were conducted by freeing selected loadings in a stepwise manner, beginning with items showing the largest (> 0.1) cross-language differences in standardized factor loadings (e.g., in order: Perceived Choice Item 4, Perceived Choice Item 2 and Values Item 7). Changes in approximate fit indices were negligible (ΔCFI ≤ 0.001; ΔRMSEA = 0.000; ΔSRMR = 0.000), and the DIFFTEST remained significant across all specifications. This pattern suggests that misfit was not attributable to a small, well-defined subset of items but rather reflected more diffuse differences across loadings. Accordingly, partial invariance did not provide a substantively clearer or more stable solution, and the more parsimonious metric specification was retained. A conventional repeated-measures CFA model was additionally estimated, allowing correlated residuals between corresponding Polish and English items. Due to extremely high cross-language latent correlations (*r* ≈ 0.94–0.99), the latent covariance matrix was numerically non–positive definite across specifications. This likely reflects very high within-person stability across languages rather than substantive misfit. Given these numerical issues and the conceptual appropriateness of explicitly modeling language as a method factor, the CT-C(M–1) model is retained as the primary basis for inference. A full correlated traits–correlated methods (CTCM) model was also estimated but did not produce a proper solution (noncomputable standard errors; near-zero condition number), indicating empirical underidentification in this high-dimensional ordinal MTMM setting. Therefore, the CT-C(M–1) parameterization was adopted.

Monte Carlo simulations were conducted for metric CT-C(M-1) model (ordinal indicators; WLSMV, theta parameterization) using 275 and 400 observations per simulated dataset and 500 replications. Parameter recovery and convergence were evaluated across replications. At *N* = 275, convergence was 21% (104 replications), at *N* = 400, convergence improved to 49% (244 replications) consistent with the pattern in single-language models and reflecting finite-sample estimation difficulties rather than structural model misspecification. Across successful replications, the median absolute parameter bias was approximately 0.011 at *N* = 275, and 0.02 at *N* = 400. Although parameter bias among converged solutions was small, the low convergence rate indicates that the CT-C(M − 1) model was close to the practical limits of the available sample size and that its parameter estimates should be interpreted with caution. The empirical CT-C(M–1) models converged without improper solutions, and the resulting substantive conclusions aligned with those obtained from simpler CFA-based analyses.

Multi-group CFAs conducted separately for the Polish and English versions across S1 and S2 supported metric invariance in both cases. Constraining factor loadings to equality across waves did not significantly worsen model fit (Polish: Δ*χ*^2^(38) = 47.53, *p* = 0.14; English: Δ*χ*^2^(38) = 38.81, *p* = 0.43), and approximate fit indices remained acceptable (Polish: CFI = 0.930, TLI = 0.934, RMSEA = 0.077, SRMR = 0.081; English: CFI = 0.928, TLI = 0.932, RMSEA = 0.076, SRMR = 0.082). These results indicate that the seven-factor structure was stable across samples in both language versions. A multi-group CT-C(M–1) analysis across S1 and S2 supported metric invariance of the language model. Constraining parameters to equality across waves did not significantly worsen model fit (Δ*χ*^2^(38) = 34.55, *p* = 0.63), and approximate fit indices indicated good fit (CFI = 0.938, TLI = 0.940, RMSEA = 0.054, SRMR = 0.086). These results support the stability of the CT-C(M–1) structure across samples and justify pooling the data for primary analyses.

Few items showed notable discrepancies in loadings between language versions. These included item 2 on the Perceived Choice subscale (ENG − 0.697 vs. PL − 0.907; difference: 0.210), item 4 on the same subscale (ENG − 0.659 vs. PL − 0.864; difference: 0.205), item 2 on the Pressure/Tension subscale (ENG − 0.687 vs. PL − 0.833; difference: 0.146), item 6 on Perceived Choice (ENG 0.981 vs. PL 0.888; difference: 0.093), and item 7 on Perceived Choice (ENG − 0.885 vs. PL − 0.793; difference: 0.092). Overall, the Perceived Choice subscale showed the most variation between languages at the item level, despite high overall cross-language correlations (*ρ* = 0.93).

One item (item 4 on Interest/Enjoyment subscale, reverse coded: “This activity did not hold my attention at all”) showed consistently poor psychometric performance across both language versions. Its item-rest correlation with the corresponding subscale was weak (0.35 for the Polish version, 0.43 for the English version, while all other item-rest correlations across subscales and language versions were at least above 0.50 and mostly above 0.70), and its standardized CFA loadings were comparatively low in both the Polish (−0.598) and English (−0.569) samples. Additionally, the item’s translation showed weak cross-language correlation (ρ = 0.40). Given this pattern, the item may warrant revision or removal in future use and adaptations. Another item with a relatively low factor loading was item 6 on the Perceived Competence subscale (“This was an activity that I could not do very well,” reverse coded) with loadings −0.528 for the Polish version and −0.616 for the English version. In this case, however, the item-rest correlations were stronger (0.52 for the Polish version, 0.54 for the English version), albeit still lower than for most other items. Cross-language correlation of this item was ρ = 0.57. Although these two reverse-worded items exhibited localized misfit across languages, the majority of reverse-worded indicators loaded as expected. This pattern suggests item-specific issues rather than a systematic wording method effect. Sensitivity analyses were conducted excluding Item 4 on Interest/Enjoyment, Item 6 on Perceived Competence, and both items across the Polish CFA, English CFA, and CT-C(M − 1) models. For both the Polish and English CFA models, the exclusion of these items resulted in virtually unchanged fit indices, with improvements in CFI not exceeding 0.01 and changes in RMSEA and SRMR remaining within approximately 0.001–0.004. In contrast, within the CT-C(M − 1) framework, removing Item 6 resulted in worse model fit (e.g., ΔCFI ≈ − 0.02, ΔRMSEA ≈ + 0.007), while other reduced models failed to converge. These findings suggest that removing weaker items does not improve model performance and may compromise the stability of the more complex multitrait–multimethod specification.

### 3.2 Descriptive statistics, normality, and reliability

Descriptive statistics and internal consistency estimates for the seven IMI subscales are presented in Table 2. All Polish subscales demonstrated acceptable to high reliability, with Cronbach’s alpha and McDonald’s omega values ranging from 0.85 to 0.93.

**Table 2:** Means, SDs, α, ω for IMI subscales.

Subscale	Polish version				English version			
	*M*	*SD*	α	ω	*M*	*SD*	α	ω
Interest/enjoyment	30.23	9.84	0.92	0.92	29.98	9.98	0.92	0.93
Perceived competence	31.14	5.96	0.85	0.86	30.59	6.30	0.87	0.88
Effort/importance	25.39	6.07	0.88	0.88	25.03	6.20	0.88	0.88
Pressure/tension	18.50	7.17	0.89	0.89	18.72	6.97	0.87	0.88
Perceived choice	26.27	10.77	0.93	0.93	25.25	10.38	0.91	0.92
Value/usefulness	33.51	9.20	0.93	0.93	33.42	8.93	0.93	0.93
Relatedness	33.74	11.25	0.93	0.93	33.93	11.46	0.93	0.93

All IMI subscales were assessed for univariate normality based on skewness and kurtosis values, Shapiro–Wilk tests, as well as visual inspection of the histograms. For both the Polish and English versions, absolute values of skewness ranged between 0.02 and 0.75, and absolute values of kurtosis ranged between 0.03 and 0.89.

### 3.3 Cross-language equivalence

Subscale scores from the Polish and English versions were strongly correlated, with values ranging from *ρ* = 0.86 to ρ = 0.94 (see Table 3). Item-level scores were also strongly correlated across language versions.

**Table 3:** Cross-language correlations between Polish and English versions of the IMI subscales.

			Overall PL-ENG Subscale correlation	
IMI subscale	Items	Item-level correlations range (Spearman’s ρ)	Spearman’s ρ [95% CI] (frequentist)	Kendall’s τ [95% CrI] (Bayesian)
Interest/enjoyment	7	0.40–0.85	0.94*** [0.93–0.95]	0.82*** [0.73–0.88]
Perceived competence	6	0.64–0.70	0.88*** [0.84–0.90]	0.73*** [0.64–0.80]
Effort/importance	5	0.59–0.77	0.86*** [0.83–0.89]	0.73*** [0.64–0.79]
Pressure/tension	5	0.63–0.74	0.90*** [0.87–0.92]	0.75*** [0.66–0.82]
Perceived choice	7	0.59–0.86	0.93*** [0.91–0.94]	0.78*** [0.69–0.85]
Value/usefulness	7	0.69–0.77	0.91*** [0.89–0.93]	0.78*** [0.69–0.84]
Relatedness	8	0.65–0.90	0.94*** [0.92–0.95]	0.81*** [0.72–0.87]

Spearman’s ρ: *p < 0.05, **p < 0.01, ***p < 0.001; Kendall’s τ: * BF₁₀ > 10, ** BF₁₀ > 30, *** BF₁₀ > 100.

The Relatedness, Interest, and Perceived Choice subscales showed the strongest overall cross-language equivalence (ρ = 0.94, *p* < 0.001; *τ* = 0.81, BF₁₀ > 100; ρ = 0.94, *p* < 0.001; τ = 0.82, BF₁₀ > 100; ρ = 0.93, *p* < 0.001; τ = 0.78, BF₁₀ > 100 respectively), while the Effort/Importance subscale showed the comparatively lowest overall cross-language correlation (*ρ* = 0.86, *p* < 0.001; *τ* = 0.73, BF₁₀ > 100). Only one item (Interest/Enjoyment Item 4) showed a notably weaker cross-language correlation (*ρ* = 0.40, *p* < 0.001; *τ* = 0.35, BF₁₀ > 100), indicating a potential issue with the translation or original conceptualization of this specific item. The next-lowest correlation on that subscale was shown by item 7 at *ρ* = 0.77 (*p* < 0.001) and *τ* = 0.69 (BF₁₀ > 100).

### 3.4 Construct validity

To assess construct validity of the Polish IMI, frequentist Spearman’s *ρ* and Bayesian Kendall’s *τ* correlations were calculated between IMI subscales and conceptually related constructs (see Table 4). The results broadly aligned with theoretical expectations. As expected, subscales conceptually closer to intrinsic engagement showed stronger associations with Attitude than did autonomy-related subscales. The pattern of associations provides preliminary support for construct validity.

**Table 4:** Convergent/divergent validity correlations of Polish IMI subscales.

Polish IMI subscale	Attitude	Perceived control	CAS–challenge	CAS–threat	CEI-II–curiosity	GSES–self-efficacy
Interest/enjoyment	0.81*** [0.77–0.85]	0.41*** [0.31–0.50]	0.56*** [0.47–0.64]	−0.24*** [−0.35–−0.13]	0.40*** [0.29–0.49]	0.36*** [0.25–0.46]
	0.65*** [0.57–0.72]	0.30*** [0.22–0.38]	0.42*** [0.33–0.49]	−0.17*** [−0.25–−0.09]	0.28*** [0.20–0.36]	0.26*** [0.20–0.36]
Perceived competence	0.51*** [0.41–0.59]	0.53*** [0.44–0.61]	0.53*** [0.44–0.61]	−0.34*** [−0.44–−0.23]	0.34*** [0.23–0.44]	0.46*** [0.36–0.55]
	0.38*** [0.30–0.46]	0.40*** [0.31–0.47]	0.40*** [0.31–0.47]	−0.24*** [−0.32–−0.16]	0.24*** [0.16–0.32]	0.33*** [0.25–0.41]
Effort/importance	0.41*** [0.31–0.50]	−0.01 [−0.13–0.11]	0.49*** [0.39–0.57]	0.17** [0.05–0.28]	0.11 [−0.04–0.23]	0.24*** [0.13–0.35]
	0.30*** [0.22–0.37]	−0.01 [−0.09–0.07]	0.35*** [0.27–0.43]	0.12 [0.04–0.20]	0.08 [−0.00–0.16]	0.17*** [0.09–0.25]
Pressure/tension	−0.34*** [−0.44–−0.23]	−0.63*** [−0.69–−0.55]	−0.12* [−0.24–−0.01]	0.61*** [0.53–0.68]	−0.23*** [−0.34–−0.11]	−0.19** [−0.30–−0.08]
	−0.24*** [−0.32–−0.16]	−0.48*** [−0.55–−0.39]	−0.09 [−0.17–−0.01]	0.45*** [0.37–0.53]	−0.16*** [−0.24–−0.08]	−0.14** [−0.22–−0.06]
Perceived choice	0.56*** [0.47–0.64]	0.41*** [0.30–0.50]	0.36*** [0.25–0.46]	−0.32*** [−0.42–−0.21]	0.30*** [0.19–0.40]	0.32*** [0.21–0.42]
	0.41*** [0.33–0.49]	0.29*** [0.21–0.37]	0.26*** [0.17–0.33]	−0.23*** [−0.31–−0.15]	0.21*** [0.13–0.29]	0.22*** [0.14–0.30]
Value/usefulness	0.80*** [0.75–0.84]	0.23*** [0.11–0.34]	0.60*** [0.52–0.67]	−0.09 [−0.21–0.03]	0.35*** [0.24–0.45]	0.40*** [0.30–0.50]
	0.63*** [0.54–0.70]	0.16*** [0.08–0.24]	0.45*** [0.37–0.52]	−0.07 [−0.14–0.01]	0.25*** [0.16–0.32]	0.28*** [0.20–0.36]
Relatedness	0.56*** [0.48–0.64]	0.34*** [0.23–0.44]	0.42*** [0.31–0.51]	−0.19** [−0.30–−0.07]	0.30*** [0.19–0.41]	0.28*** [0.17–0.39]
	0.41*** [0.33–0.49]	0.25*** [0.17–0.32]	0.30*** [0.21–0.37]	−0.13* [−0.21–−0.06]	0.22*** [0.14–0.29]	0.20*** [0.12–0.28]

Shown correlations represent frequentist Spearman’s ρ (95%CI) (upper part of the row) and Bayesian Kendall’s τ (95%CrI) (lower part of the row) coefficients between IMI subscales in Polish and other constructs. Spearman’s ρ: *p < 0.05, **p < 0.01, ***p < 0.001; Kendall’s τ * BF₁₀ > 10, ** BF₁₀ > 30, *** BF₁₀ > 100.

Subscales most directly reflecting task involvement — Interest/Enjoyment, Effort/Importance, Value/Usefulness — showed similar correlation patterns with other constructs, mainly with Attitude, Challenge, and Curiosity. Interest/Enjoyment showed strong positive correlations with Attitude (*ρ* = 0.81, *p* < 0.001; *τ* = 0.65, BF₁₀ > 100) and Challenge (ρ = 0.56, *p* < 0.001; τ = 0.42, BF₁₀ > 100), supporting convergent validity as a measure of intrinsic motivation. It also correlated with Curiosity (*ρ* = 0.40, *p* < 0.001; *τ* = 0.28, BF₁₀ > 100). A similar pattern was observed for Effort/Importance, which correlated positively with Attitude (ρ = 0.41, *p* < 0.001; τ = 0.30, BF₁₀ > 100) and Challenge (ρ = 0.49, *p* < 0.001; τ = 0.35, BF₁₀ > 100) as expected for a measure of engagement. It also correlated weakly with Threat (*ρ* = 0.17, *p* < 0.01; *τ* = 0.12, BF₁₀ = 6.61), probably reflecting mobilization under stress, although Bayesian result is inconclusive while frequentist one is significant. Value/Usefulness was also most strongly related to Attitude (*ρ* = 0.80, *p* < 0.001; *τ* = 0.63, BF₁₀ > 100), in line with its role as a cognitive appraisal of task relevance. It was also positively correlated with all other scales except Threat.

Three subscales related to autonomy and agency — Perceived Choice, Pressure/Tension, and Perceived Competence — also showed similar patterns, correlating with Perceived Control and Self-efficacy. As expected, Pressure/Tension correlated negatively with Attitude, Perceived Control, Curiosity, and Self-efficacy, while showing a strong positive correlation with Threat (*ρ* = 0.61, *p* < 0.001; *τ* = 0.45, BF₁₀ > 100), reflecting divergent validity. Perceived Choice was positively correlated with Attitude, Perceived Control, Challenge, Curiosity, and Self-efficacy, confirming its links to autonomy-related processes. It was also negatively related to Threat (ρ = −0.32, *p* < 0.001; τ = −0.23, BF₁₀ > 100), consistent with the notion that voluntarily chosen tasks are more likely to be appraised as challenging than threatening. Perceived Competence was most strongly associated with Attitude (ρ = 0.51, *p* < 0.001; τ = 0.38, BF₁₀ > 100), Perceived Control (ρ = 0.53, *p* < 0.001; τ = 0.40, BF₁₀ > 100), Challenge (ρ = 0.53, *p* < 0.001; τ = 0.40, BF₁₀ > 100), and Self-efficacy (ρ = 0.46, *p* < 0.001; τ = 0.33, BF₁₀ > 100), consistent with its intended overlap with agency-related constructs.

Relatedness followed a pattern similar to most other subscales, being most strongly related to Attitude (ρ = 0.56, *p* < 0.001; τ = 0.41, BF₁₀ > 100), with lower correlations with other scales and a negative one with Threat.

## 4 Discussion

This adaptation provides a full Polish version of the Intrinsic Motivation Inventory (IMI) with strong reliability, robust cross-language correspondence, and approximate metric invariance across Polish and English in a pooled bilingual sample. Taken together, the findings support approximate metric equivalence in bilingual adults under within-person conditions and the Polish version of the IMI appears suitable for Polish-language research as well as in cross-language comparisons.

### 4.1 Structural validity and invariance

Seven correlated factors fit both language versions acceptably. In the pooled CT-C(M–1) framework, the marker-configural model already showed acceptable fit. Constraining to a strict metric specification produced only minimal changes in approximate fit indices (ΔCFI = +0.005; ΔRMSEA = −0.002; ΔSRMR = +0.002), well within common guidelines for metric invariance. As expected with WLSMV, DIFFTEST was significant; however, given the model size and sample, the approximate-fit deltas provide the more informative evidence. This pattern supports comparing covariances/associations across languages and suggests that item–trait relations are functionally equivalent. Wave-level checks further indicated that the language-metric structure was stable across S1 and S2, justifying the pooled analyses.

The overall CFA results broadly align with reports in other languages, although many prior adaptations used shortened forms (fewer items, subscales, or factors). Better fit in those cases may reflect the reduced model complexity. The original study by McAuley et al. (1989) reported CFA on 18 items spanning 4 subscales (plus one second-order factor), whereas the present adaptation translated the full 45 items across 7 subscales.

### 4.2 Item-level observations

Most items loaded strongly on their intended factors. Two items, however, warrant particular attention. Interest/Enjoyment item 4 showed comparatively low loadings in both languages, weak item–rest correlations, and a low cross-language correlation. Because this pattern was observed consistently across Polish and English versions, it likely reflects characteristics of the item’s reverse wording and attentional framing rather than translation-specific issues. Perceived Competence item 6 also showed comparatively lower loadings, although its item–rest correlations were acceptable.

Sensitivity analyses excluding these items indicated that their removal did not meaningfully improve model fit in the Polish or English CFA models, and led to instability or poorer fit in the CT-C(M − 1) model. This suggests that, despite their weaker individual performance, these items contribute to the overall structure of the scale. Accordingly, all items were retained to preserve comparability with the original instrument. However, given their relatively weaker psychometric properties, these items should be interpreted with caution and may be considered potentially problematic in Polish samples. Future research should continue to monitor their performance and consider revision or removal if similar patterns are consistently observed.

### 4.3 Internal consistency and cross-language correlations

Internal consistency (Cronbach’s *α* and McDonald’s *ω*) was high across all subscales in both the Polish and English versions, ranging from 0.85 to 0.93 and indicating that the Polish translation preserves the psychometric robustness of the instrument. Reliability is therefore comparable, or even better, with adaptations to other languages (despite structural variations, other languages adaptations’ subscales demonstrate good internal consistency, with Cronbach’s α values ranging from 0.70 to above 0.90). Cross-language correlations (original and translated versions administered to Polish bilingual participants) were also high, supporting the functional equivalence of the Polish version.

### 4.4 Construct validity

Construct validity was supported by correlations of IMI subscales with theoretically relevant constructs, in line with expectations. Because there is no general intrinsic motivation scale currently available in Polish (existing measures typically targeting specific domains such as academic context; e.g., Ardeńska et al., 2019), we chose comparison scales expected to correlate with the IMI subscales in distinct ways, avoiding redundancy.

As expected, Interest/Enjoyment correlated strongly with Attitude (Ajzen, 1991), Challenge stress appraisal (Skinner and Brewer, 2002), and Curiosity (Kashdan et al., 2009) consistent with its central role as indicator of intrinsic motivation. Another scale linked to task engagement, Effort/Importance, also correlated with Attitude and Challenge, and showed a weak but significant association with Threat stress appraisal (Skinner and Brewer, 2002), potentially reflecting mobilization in demanding, personally important contexts. It is consistent with SDT’s distinction between autonomous and controlled forms of motivation (Ryan and Deci, 2000). However, this association was inconclusive in Bayesian terms and should be interpreted cautiously. Value/Usefulness showed a similar pattern to Interest/Enjoyment, also correlating highly with Attitude, reflecting its role as a cognitive appraisal of task relevance. Value/Usefulness is conceptually close to evaluative attitudes toward the task, so a high association is expected and supports convergent validity. Subscales related to autonomy and competence (Perceived Competence and Perceived Choice) correlated with Perceived Control (Ajzen, 1991), Self-Efficacy (Schwarzer and Jerusalem, 1995), and Challenge. These are constructs grounded in agency, autonomy, and perceived capability. Pressure/Tension showed expected inverse pattern, correlating negatively with all adaptive motivation-related constructs and positively with Threat, supporting its divergent validity. Relatedness correlated primarily with Attitude; this subscale would benefit most from additional construct-validity work, including more external criteria for validation.

### 4.5 Limitations

This study has several limitations. First, the final pooled sample consisted primarily of young adults recruited via an online platform and thus reflects a relatively educated, digitally literate population typical of Prolific samples. While this sampling approach is common in psychometric research and appropriate for initial validation, it does not yield a representative sample of the general Polish population. In particular, older individuals, less digitally engaged groups, and individuals with lower levels of education may be underrepresented. Accordingly, the present findings should be interpreted as most directly generalizable to similar online, bilingual populations, and further validation in more diverse samples is warranted.

Regarding sample size, larger and more diverse samples would allow more precise parameter estimation and more demanding invariance testing. No formal *a priori* power analysis was conducted, and the sample size should be considered borderline given the complexity of the estimated models. Sample size requirements in CFA and SEM depend on model complexity, indicator quality, factor loadings, and model identification rather than a single universal threshold (Kline, 2016; Wolf et al., 2013). Although the sample size was adequate for estimating the primary CFA models, the Monte Carlo simulations indicated reduced convergence at *N* = 275, particularly for the CT-C(M − 1) model. This suggests that the more complex multitrait–multimethod specification may be underpowered or overparameterized relative to the available data. Accordingly, results from the CT-C(M − 1) model should be interpreted as supportive but preliminary, and future studies should replicate the model in larger samples.

Construct validity was only partially assessed, and the set of external scales was limited. Importantly, the study did not include a direct criterion measure of intrinsic motivation, which constrains the strength of the validity evidence. While the selected measures were theoretically relevant, future studies should include validated domain-specific measures of intrinsic motivation (e.g., WEIMS-PL; Academic Motivation Scale), as well as behavioral indicators (e.g., free-choice persistence) to strengthen the evidence base, especially for domain-specific applications (e.g., academic, school, work, sport). Especially for domain-specific applications (e.g., academic, school, work, sport). This would also improve comparability with prior validation studies in other languages.

The within-person administration of both language versions may have introduced carryover effects, including memory or consistency biases and demand characteristics that could have influenced participants’ responses across versions. At the same time, this design reduces between-person variability and thus provides a more controlled comparison of the two language versions. These features represent a trade-off: while within-person administration enhances comparability, it may also lead to participants being inclined to respond consistently across versions or in line with perceived study expectations, particularly given the similarity of item content. As a result, the observed cross-language associations could be inflated and should be interpreted as potentially reflecting an upper-bound estimate of linguistic equivalence. Although randomization and anonymity were employed to mitigate such effects, future studies could more directly address this issue by introducing temporal separation between language administrations or by using independent samples. As with most self-report instruments, responses may also be influenced by social desirability and common-method variance, shared response format across scales and same-session measurement of all constructs, which could also somewhat inflate correlations. Future studies may benefit from multi-method designs or behavioral validation tasks.

Finally, the task instruction asked participants to reflect on a single, self-selected collaborative activity completed with someone they did not know well. This represents a notable limitation of the present study, as this specific context may have differentially activated certain subscales, potentially inflating scores on some (e.g., Relatedness) while restricting variability in others (e.g., Perceived Choice). Consequently, the generalizability of the obtained subscale means and intercorrelation patterns to other types of activities may be limited. Future research should therefore examine the measurement properties of the scale across a broader range of task contexts, including independent work, competitive settings, and formal learning environments.

### 4.6 Future directions

To strengthen the psychometric evidence for the Polish IMI, future work should recruit larger, more diverse samples spanning different regions, age groups, and occupational or educational contexts. Testing in monolingual Polish samples, outside Prolific/online context and in non-young adult populations would allow for checking measurement invariance across demographic and cultural subgroups, increasing confidence in the generalizability of findings.

Future research could also examine associations between the Polish IMI and domain-specific motivational measures within clearly defined contexts (e.g., academic or occupational settings), to further delineate state–trait correspondences.

Longitudinal designs would allow tests of temporal stability of IMI subscales and their sensitivity to change (e.g., by intervention), which would be essential for applied purposes such as clinical interventions and training evaluations. The adaptation should also be examined across varied contexts, such as clinical, organizational, sport, and age groups to evaluate broader applicability and cultural flexibility.

## Data Availability

The datasets analyzed and Mplus, JASP and jamovi files with code and results for this study can be found in the OSF https://doi.org/10.17605/OSF.IO/CSH38. The full Polish version of IMI is available on Center for Self-Determination Theory at: https://selfdeterminationtheory.org/ consistent with how the original English version is distributed. Further inquiries can be directed to the corresponding author.
